# Factors associated with one year retention to methadone maintenance treatment program among patients with heroin dependence in China

**DOI:** 10.1186/1747-597X-9-11

**Published:** 2014-02-24

**Authors:** Haifeng Jiang, Yun Han, Jiang Du, Fei Wu, Ruimin Zhang, Huaihui Zhang, Jun Wang, Zhirong Zhou, Yih-Ing Hser, Min Zhao

**Affiliations:** 1 Shanghai Mental Health Center, Shanghai Jiao Tong University School of Medicine, Shanghai 200030, China; 2 University of Michigan, Ann Arbor, MI, USA; 3 University of California, Los Angeles, CA, USA; 4 Yunnan Institute on Drug Abuse, Kunming, Yunnan, China; 5 Shanghai Yangpu District Mental Health Center, Shanghai, China; 6 Shanghai Xuhui District Mental Health Center, Shanghai, China; 7 UCLA Integrated Substance Abuse Programs, 1640 Sepulveda Blvd., Los Angeles, CA 90025, USA

**Keywords:** Heroin dependence, Methadone maintenance treatment, Survival analysis

## Abstract

**Objective:**

The aim of this study was to evaluate the risk factors associated with dropout from Methadone Maintenance Treatment (MMT) clinics within a 1 year follow-up cohort study in China.

**Methods:**

A data analysis is to explore the adherence of MMT during one year from three hundred and twenty patients with heroin dependence at five clinics (3 in Shanghai, 2 in Kunming) in China. All participants were from the part of China-United States cooperation project entitled “Research about improving the compliance and efficacy of methadone maintenance treatment in China”. Our data analysis includes the patients’ attendance in the 6 months clinical study and the data in another 6 months afterward. The data of patients at baseline were collected with the Addiction Severity Index (ASI) which is a semi-structured questionnaire covering socio-demographic characteristics and drug use history. The one year attendance after recruitment at the clinics and daily dose were abstracted from the MMT clinic register system. The Cox proportional hazards model were used to explore the risk factor of dropout, defined as seven consecutive days without methadone.

**Results:**

By the end of 1 year of treatment 86 patients still remained in MMT without dropout (87% in Shanghai and 13% patients in Kunming). Over the entire 1-year period the median days of remaining in the program were 84 days (in Shanghai and Kunming were 317 days and 22 days).The factors associated with retention included age (HR = 0.98, 95%C.I.:0.96-0.99, P = 0.0062) and ASI alcohol scores (HR = 5.72, 95%C.I.:1.49-21.92, *P* = 0.0109) at baseline.

**Conclusion:**

One year retention of newly recruited patients with heroin dependence was related to age and ASI alcohol scores at baseline. The adherence is poorer for the patients who are young and having more serious alcohol problems.

## Introduce

According to the latest Annual Report on Drug Control in China, heroin continues to be the main drug for drug use in China: 1.156 million of the 1.794 million drug dependents registered by the national public security system in China (64.5%) were addicted to heroin [1].

In order to address this problem, especially strengthen HIV/AIDS prevention and control efforts among intravenous drug users, the government established a national working group to manage a nationwide community-based Methadone Maintenance Treatment (MMT) program for patients with deroin dependence, including 719 clinics in 28 provinces by the end of 2011 [1]. MMT program was developed rapidly during these years and 337,000 patients with deroin dependence have received MMT service since the program started in March 2004. However, only 134,000 patients have been stabilized in treatment [2]. Even in Shanghai, the most developed city in China, the MMT program (which started in September 2006) includes 14 clinics and have treated a total of 5 000 patients, only 2 000 of which had been stabilized on daily methadone maintenance. Previous studies also show that the MMT clinics face a lot of problems that the coverage of the MMT program is quite limited and the retention of individuals in the program is poor [2].

There are numerous evidences from previous researches and program evaluations that different socio-economic status, drugs history may affect the MMT treatment outcomes. A study in Yunnan Province revealed that patients’ early dropout from MMT was related to ethnicity, clinic accessibility, living with drug users and methadone dose [3]. Whereas another study showed age, relationship with family, live on support from family or friends, income, considering treatment cost suitable, considering treatment open time suitable, addiction severity (daily expense for drug), communication with former drug taking peer, living in rural area, daily treatment dosage, sharing needles, re-admission and history of being arrested were predictors for MMT retention in Pearl River Delta, China [4]. Similar researches in different regions of China showed that the results vary among different study population [5-7]. We undertook the present data analysis to evaluate the risk factors associated with dropout from MMT clinics during one year follow-up in China. The results may be conducive to determine the role of treatment in the achievement of improved outcomes, as well as identify barriers to MMT.

## Participants and method

This data analysis is to explore the adherence of MMT during one year from 320 participants. All participants were from the part of China-United States cooperation project entitled “Research about improving the compliance and efficacy of methadone maintenance treatment in China” and approved by the ethics committee of the University of California at Los Angeles (UCLA IRB #: G08-03-087-01) where all participants received MMT for the first 24 weeks while half of them combined contingency management (CM) in the first 12 weeks to test the effect of CM in the improve of MMT adherence. CM uses behavioral reinforcement techniques based on Skinner’s Operational Conditional Reflex theory and “reinforced contingency” principle. The result of that project showed that CM had statistically significant impact on improves of MMT adherence by 24 weeks only in part of study participants. After the project, the participants continued to receive MMT. Our analysis includes the data in those 24 weeks and the data in another 6 months afterward.

### Participants

The study sample consisted of 320 patients newly recruited at five MMT clinics (3 in Shanghai, 2 in Kunming) from April 2009 to January 2010. The data at baseline from the same participants in another published study assessing the efficacy of contingency management (CM) [8] were used in this study. All participants met the following criteria: provided written informed consent, met DSM-IV criteria of heroin dependence, had no other serious mental disorders and had not received MMT treatment in the last six months.

### Data collection procedure

Patients who newly recruited in the MMT clinic were required to receive standard MMT. The standardized MMT included daily visits to the clinic to take oral methadone solution at the dosage determined by the doctors who worked in that clinic (based on the client’s condition), regular urine and blood testing. The cost of this treatment was 10 Chinese Yuan per visit which cannot be covered or reimbursed by the civil health insurance in China. Therefore, the patients had to pay this fee by themselves. If there is no medication for 7 consecutive days, the patient will be deemed to automatically dropout from treatment. If a patient return to MMT after dropout, he/she will be treated as a new start. The patients’ medical information including the attendance to the clinic and that day’s dosage of methadone was recorded into the MMT clinic register system, an online software supervised by Yunnan Institute on Drug Abuse.

All newly recruited patients were randomly assigned to receive standard MMT or standard MMT supplemented by a 12-weeks Contingency Management intervention.Participants 12 weeks after the end of the intervention were continue to receive their regular MMT treatment after the end of the intervention. At the baseline, a Chinese translation of the Addiction Severity Index (ASI) that has good reliability and validity in China [9,10] was used to assess the social demographic characteristics and history of drug use. Staffs who worked in each clinic were trained by the principal investigator (PI) and conducted face-to-face interviews using the ASI. The PI monitored or supervised them throughout the data collection process. The one year medical information after enrollment of all participants was exported from each MMT clinic register system. ‘Retention’ was defined as the number of days from first methadone uptake to dropout during the 1 year (365 days), and ‘Dropout’ was defined as seven consecutive days without methadone.

### Data analysis

Group differences between ‘dropout’ drug users and non-dropout drug users were tested using t-test and Chi-square test for continuous variables and categorical variables respectively. The group comparison included patients’ characteristics, drug use history, and ASI score at baseline. Survival analysis was used to compare treatment retention between Shanghai and Kunming sites. We conducted Cox regression survival analysis to detect potential risk factors for delay getting treatment or drop out of treatment. An event was considered to have occurred when a patient dropped out of treatment (no methadone for 7 consecutive days), and data were censored if the patient did not have an event at the end of one year in our study. Demographic variables (age, gender, ethnicity, education, employment and married status, treatment site etc.), drug use history variables (age of first drug use, primary drug, average time of drug use before entering MMT, etc.), ASI scores at baseline, Methadone dosage, and whether being in treatment group were predictors in the survival analysis. An interaction term of site and whether to accept CM intervention was included since we hypothesized that the effect of intervention varied by treatment sites. The backward selection technique was used to get the reduced model. Under this approach, we started with fitting a model with all the variables of interest, and dropped the least significant variable which was not significant at our chosen critical level, p > 0.05. We continually refit the reduced models with the same rule until all remaining variables are statistically significant. Adjusted Hazard ration with 95% confident intervals were presented in this study. Analyses were conducted using SAS 9.3 (Cary, NC, USA).

## Results

### Characteristics of the participants

The basic characteristics of the 320 enrolled participants are shown on Table 1. Almost 76.1% were male. The most common ethnicity was Han (95.27%), approximately half of the patients were unmarried and unemployed and most of them only had education level of high school and below. They had started abusing drugs in their late twenties and had a 10.72 year history of abuse on average.

**Table 1:** Background characteristics and drug use of patients by censor at baseline, % or mean (S.D.)

**Characteristics**	**Non dropout (N = 86)**	**Dropout (N = 233)**	**Total (N = 319)**	**Df**	**t value/Chi-square**	**P-value**
Age **	40.42(9.30)	36.72(8.39)	37.71(8.77)	314	2.91	0.0039
Age of first use*	28.22(7.71)	26.21(7.04)	26.75(7.27)	312	2.2	0.0284
Average drug use times before entering MMT	3.58(1.56)	3.37(1.62)	3.44(1.61)	312	1.02	0.3064
Average drug use amount before entering MMT	0.76(0.40)	0.71(0.55)	0.72(0.51)	310	0.75	0.454
Dosage at baseline	30.26(13.46)	32.94(18.80)	32.23(17.55)	297	−1.16	0.2469
Surround drug users	1.77(2.80)	1.38(2.78)	1.66(2.79)	313	−1.5	0.134
Gender: Male(%)	67(77.91)	176(75.43)	243(76.1)	1	0.2115	0.6456
GROUP				1	0.9514	0.3294
MMT + CM, n(%)	47(54.65)	113(48.50)	160(50.16)			
MMT only, n(%)	39(45.35)	120(51.50)	159(49.84)			
Place***				1	65.7585	<.0001
Kunming, n(%)	11(12.8)	149(63.95)	160(50.16)			
Shanghai, n(%)	75(87.2)	84(36.05)	159(49.84)			
Education				1	0.329	0.5663
Highschool and below, n(%)	85(98.48)	228(97.84)	313(98.12)			
College and above, n(%)	1(1.16)	5(2.16)	6(1.88)			
Employment				2	4.6246	0.099
Not in labor force, n(%)	3(3.49)	4(1.72)	7(2.19)			
Unemploymed, n(%)	53(61.63)	119(50.86)	172(53.61)			
Employed, n(%)	30(34.88)	110(47.41)	140(44.2)			
Race				1	1.458	0.2272
Han, n(%)	84(97.65)	220(94.4)	304(95.27)			
Others, n(%)	2(2.35)	13(5.6)	15(4.73)			
Living with someone with current alcohol problem						
No(%)	98.84	96.55	97.17	1	1.1917	0.275
Living with someone who uses or abuses drugs						
No(%)	91.86	91.38	91.51	1	0.0187	0.8912
Living status				2	4.869	0.0876
Dependent, n(%)	72(83.72)	170(72.84)	242(75.86)			
Independent, n(%)	14(16.28)	58(25)	72(22.57)			
Others, n(%)	0(0.00)	5(2.16)	5(1.57)			
Primary drug				3	7.0797	0.0694
No, n(%)	3(3.49)	7(3.03)	10(3.15)			
Heroin, n(%)	52(60.47)	175(75.32)	127(71.29)			
Alcohol with one or more drugs, n(%)	9(10.47)	15(6.49)	24(7.57)			
Two or more drugs without alcohol, n(%)	22(25.58)	35(15.15)	57(17.98)			
Marital status				2	1.6219	0.4444
Never married, n(%)	44(51.16)	104(44.83)	148(46.39)			
Married/Remarried, n(%)	27(31.4)	90(38.79)	117(36.99)			
Divorce/Windowed, n(%)	15(17.44)	39(16.38)	54(16.61)			
ASI score at baseline						
Medical status composite score	0.13(0.19)	0.13(0.22)	0.13(0.22)	303	0.00	0.9962
Employment status composite score***	0.55(0.25)	0.5(0.29)	0.52(0.28)	305	5.09	<0.0001
Alcohol use composite score	0.03(0.08)	0.06(0.11)	0.05(0.11)	295	−1.81	0.0717
Drug use composite score	0.17(0.10)	0.2(0.10)	0.19(0.10)	303	−2.45	0.0148
Legal status composite score***	0.15(0.14)	0.08(0.11)	0.1(0.12)	284	4.35	<.0001
Family status composite score	0.15(0.17)	0.11(0.13)	0.12(0.15)	297	0.29	0.7723
Psychiatric status composite score	0.06(0.12)	0.05(0.13)	0.06(0.12)	308	0.44	0.6585

Chi-square test or t-test on group differences. *p < 0.05, **p < 0.01, ***p < 0.001.

Despite the random assignment to groups, there were differences observed across sites that a lower dropout rate in the patients in Shanghai than those in Kunming. Participants dropped out from MMT were more likely to be younger or have early onset of heroin use than those remain in treatment. The mean average daily MMT dose were 30.26(13.46) mg in non-dropout group and 32.94(18.80) mg in dropout group, but comparison revealed no significant difference between the two groups.

### Retention in MMT treatment for one year

**Figure 1:**
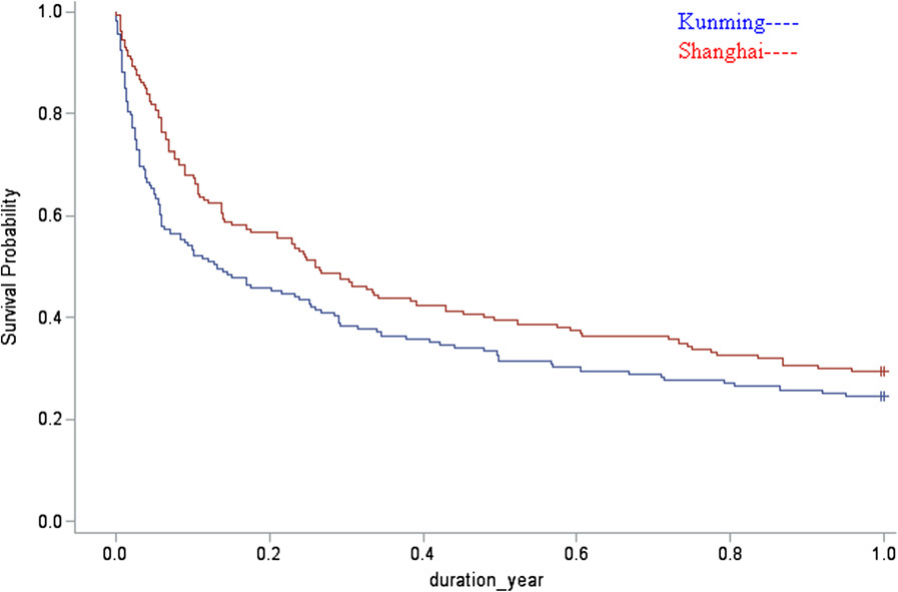
**The survival curve for Shanghai and Kunming.** (n = 319).

By the end of 1 year of treatment 86 patients still remained in MMT without dropout (87% in Shanghai and 13% patients in Kunming). Over the entire 1-year period the median days of remaining in the program were 84 days (in Shanghai and Kunming were 317 days and 22 days). By the end of 1 year of treatment, 47 participants (45.35%) in the MMT group and 39 subjects (54.65%) in the MMT + CM group remained in treatment (Chi-sqr = 0.78, df = 1, p = 0.3771, Cramer’s V = 0.0565). Over the entire 1-year period the median days of remaining in the program in the MMT and MMT + CM groups were 135 days and 164 days, respectively (Z = 10.2426, p < 0.001, df = 1) (Figure 1).

### Cox regression model results

Table 2 presents the findings from the stepwise multiple Cox regression predicting treatment retention. After adjusting for intervention, treatment place, and the interaction of treatment place and intervention in the model, dropout was more likely to happen among young patients than elderly patients. The other variable was ‘ASI alcohol scores’, dropout was more likely to occur in the patients with more serious alcohol problems than the patients having less alcohol problems.

**Table 2:** Cox regression predicting treatment retention with participants entering treatment

**Predictor**	**Adjusted hazard ratio**	**95% hazard ratio confidence limits**		**DF**	**Wald Chi-Square**	***p***
Alcohol composite score at baseline	5.721	1.493	21.924	1	6.476	0.0109
Age	0.977	0.961	0.993	1	7.4882	0.0062

## Discussion

To the best of our knowledge, this is the first study in China to report the factors associated with retention to Methadone Maintenance Treatment program among patients with deroin dependence over a period of one year. Two factors, namely age and ASI alcohol scores, were found to significantly predict retention in the MMT program.

This study was conducted during the 2010 Shanghai World Exposition when public security highly monitored drug users so that the patients had less chance to access to heroin and all the participants in Shanghai were being closely supervised by anti-drug social workers, which might be the factors that may have temporarily increased MMT clinic retention rate in Shanghai. A previous study conducted in Yunnan Province of China indicated that the cumulative probability of retention at 1, 3 and 6 months was 94%, 75% and 57% [3]. But the retention rate by the end of 1 year in our study decreased to 50% on top. Despite a rapid and substantial expansion of MMT program coverage in China, the retention of individuals getting into the MMT programs is still poor especially with the time extension of maintenance therapy.

This study found out that one factor related to patients’ retention was ‘Age’. Our study results successfully replicated western [11] and local [4] studies that older patients had better outcomes regardless of the clinic setting. Previous qualitative studies [12] suggested that the marginalization of older heroin dependences was a predominant experience that impacted the intent to seek treatment as well as treatment retention.

The strongest predictor of dropout was ‘ASI alcohol scores’. When each score increases in ‘ASI alcohol scores’ at baseline, clients were 5.72 times more likely to dropout from treatment during 1 year from recruitment. Our study result that patients with more pre-treatment alcohol problem may have more risk to dropout from MMT seems to be globally comparable with previous studies [13,14]. The cause might be that drinking problem among the patients undergoing MMT is associated with an increased risk of relapse into other illegal drugs [15]. Whereas, alcohol is an enzymatic inductor of methadone catabolism, intoxication or lethal events can occur due to additive neuro-depressant effects which may cause interruption of MMT [16]. Thus a substitution assessment/intervention is recommended among the patients with drinking problem during MMT.

There were several limitations to this study. Firstly, we should note that multiple testing on group deference of demographic and drug use history between dropout patients and non-dropout patients comprises multiple significance testing, thereby inflating Type I error. Secondly, all participants were only recruited from two cities in China, and from two kinds of settings, 3 clinics from an urban setting (Shanghai is the biggest modern city in China) and 2 clinics from a rural setting (Kunming, which is the capital city of Yunnan Province, which its long borders with the infamous Golden Triangle). Patients from two kinds of setting differ in financial situation, monitoring system being in and other living situations. However, the effects of socio-economic status and supervision by social workers during the study period were not revealed, which may associated with outcomes of MMT [17,18]. Thirdly, there was potential effect due to facing emerging issues (2010 World Expo was held in Shanghai) that may affect the study results. With the above mentioned limitations, the results should be explained in caution and may not be applicable to the other MMT patients in China.

## Conclusion

Government had taken a lot of efforts to run the MMT program, however, the program has a poor retention rate. Age and severity of alcohol use were the risk factors associated with retention of one year after enrollment in MMT program, our study suggests that developing intervention for patients who are young and with drinking problem during MMT treatment is recommended, which may improve the retention and efficacy of MMT.

## Competing interest

The authors declare that they have no competing interests.

## Authors’ contributions

MZ and YH designed the study. HJ, JD, FW, RZ, HZ, JW and ZZ conducted the research. HJ and YH conducted the data analysis. HJ and YH prepared the manuscript. All authors read and approved the final manuscript.
